# The Impact of Whaling on the Ocean Carbon Cycle: Why Bigger Was Better

**DOI:** 10.1371/journal.pone.0012444

**Published:** 2010-08-26

**Authors:** Andrew J. Pershing, Line B. Christensen, Nicholas R. Record, Graham D. Sherwood, Peter B. Stetson

**Affiliations:** 1 School of Marine Sciences, University of Maine, Orono, Maine, United States of America; 2 Gulf of Maine Research Institute, Portland, Maine, United States of America; 3 Fisheries Centre, University of British Columbia, Vancouver, British Columbia, Canada; University of Hull, United Kingdom

## Abstract

**Background:**

Humans have reduced the abundance of many large marine vertebrates, including whales, large fish, and sharks, to only a small percentage of their pre-exploitation levels. Industrial fishing and whaling also tended to preferentially harvest the largest species and largest individuals within a population. We consider the consequences of removing these animals on the ocean's ability to store carbon.

**Methodology/Principal Findings:**

Because body size is critical to our arguments, our analysis focuses on populations of baleen whales. Using reconstructions of pre-whaling and modern abundances, we consider the impact of whaling on the amount of carbon stored in living whales and on the amount of carbon exported to the deep sea by sinking whale carcasses. Populations of large baleen whales now store 9.1×10^6^ tons less carbon than before whaling. Some of the lost storage has been offset by increases in smaller competitors; however, due to the relative metabolic efficiency of larger organisms, a shift toward smaller animals could decrease the total community biomass by 30% or more. Because of their large size and few predators, whales and other large marine vertebrates can efficiently export carbon from the surface waters to the deep sea. We estimate that rebuilding whale populations would remove 1.6×10^5^ tons of carbon each year through sinking whale carcasses.

**Conclusions/Significance:**

Even though fish and whales are only a small portion of the ocean's overall biomass, fishing and whaling have altered the ocean's ability to store and sequester carbon. Although these changes are small relative to the total ocean carbon sink, rebuilding populations of fish and whales would be comparable to other carbon management schemes, including ocean iron fertilization.

## Introduction

Ecosystems play a central role in regulating the concentration of atmospheric carbon dioxide. Whether an ecosystem acts as a source or sink for atmospheric carbon dioxide depends on the relative rates of photosynthesis and respiration. Research on the organic carbon cycle in the ocean has focused on the processes that limit primary productivity and the removal of carbon from the euphotic zone through the biological pump [1]. Primary production in the ocean is limited by the availability of macronutrients (principally, nitrogen) and micronutrients, notably iron [2]. On annual or longer scales, the availability of these nutrients is controlled by physical processes such as vertical mixing, upwelling, or atmospheric deposition. Carbon is removed from the euphotic zone by the sinking of organic matter. Dead phytoplankton cells are the dominant component of the flux, but zooplankton feces and vertical migrations can contribute significantly [1]. On land, humans directly influence the carbon stored in terrestrial ecosystems through logging and the burning of forests and grasslands. In the open ocean, the carbon cycle is assumed to be free of direct human influences [3].

Humans have had a substantial impact on the abundance, biomass, and size structure of populations of fish and whales [4], [5], [6]. Yet, due to their low abundance relative to plankton, marine vertebrates are not included in most models of marine biogeochemistry [7], [8], [9]. Although only a small part of the ocean's total carbon budget, marine vertebrates contribute to the movement and storage of inorganic [10] and, as we will show, organic carbon. These species may also influence the availability of micronutrients such as iron [11]. The nature of the exploitation, which focused on the largest individuals and species, further magnified the impact of fishing and whaling on the carbon cycle due to the inherent metabolic efficiency of large animals. As considerations of body size are central to our calculations, our analysis will focus on baleen whales, although our arguments also apply to fish and sharks.

## Results

**Figure 1:**
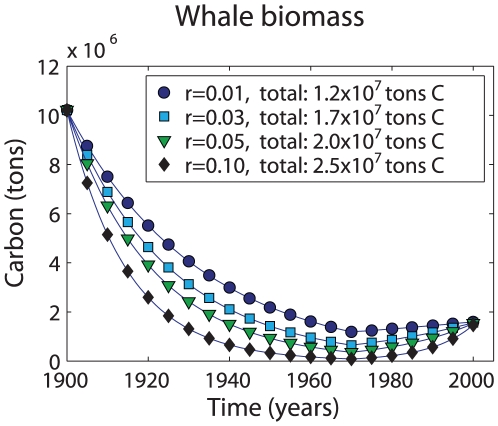
Biomass of eight species of large whales. Each line represents a different biomass accumulation rate (*r*) as indicated in the key. Each *r* implies a distinct level of whaling in order to reach the specified 2001 biomass levels. The total biomass of whales that must be removed for each *r* is also indicated in the key.

Compared to phytoplankton that have life spans measured in days, whales and large fish live for many decades. The carbon accumulated in the body of a long-lived vertebrate will remain out of the atmosphere for the animal's life. In terms of their size and potential to store carbon for years or decades, marine vertebrates are the only organisms in the ocean comparable to large trees. Industrial whaling has largely ceased, however, the biomass of whales is less than 25% of pre-whaling levels [6], [12] (Table 1). The impact of whaling was even more catastrophic for specific populations. For example, blue whales in the Southern Ocean have been reduced by more than 99% [6]. Assuming a population recovery rate of 3% yr^−1^ and the pre- and post-whaling biomasses in Table 1, we estimate that whaling removed 1.7×10^7^ tons of carbon from marine ecosystems (Figure 1). The total carbon removed depends on the value of *r* used. Stock assessments for many populations of large whales suggest that most populations are recovering at rates around 6%, but values over 10% have been reported [13]. Using a higher *r* increases the total carbon removed.

**Table 1:** Pre-whaling and modern (2001) abundance and biomass of 8 species or species groups of baleen whales (blue = *Balaenoptera musculus*, fin = *B. physalus*, humpback = *Megaptera novaeangliae*, sei/Bryde'*s = B. borealis* and *B. brydei*, minke = *B. acutorostrata* and *B. bonaerensis*, gray = *Eschrichtius robustus*, right = *Eubalaena* spp., bowhead = *Balaena mysticetus*) from [12].

	Abundance		Biomass (tons)		Gross Flux (tons C ind^−1^yr^−1^)	Export (tons C/yr)	
Species	Pre-whaling	2001	Pre-whaling	2001		Pre-whaling	2001
Blue	340,280	4,727	35,730,693	496,353	0.424	72,172	1,003
Fin	762,400	109,600	43,339,848	6,230,387	0.223	85,180	12,245
Humpback	231,700	42,070	6,151,172	1,116,874	0.103	11,890	2,159
Sei/Bryde's	392,300	181,490	6,566,730	3,017,572	0.424	12,037	5,540
Minke	637,000	506,900	5,060,496	4,099,570	0.018	8,525	6,906
Gray	24,600	15,936	674,466	436,922	0.105	1,287	834
Right	84,100	9,239	3,074,915	337,802	0.137	1,156	127
Bowhead	89,000	9,450	2,420,141	256,970	0.051	455	48
Total	2,561,380	879,412	103,018,460	15,992,451		192,702	28,862
Change	−1,681,968		−87,026,010			−163,840	

An age-structured model was built for each species group and was used to estimate the stable age distribution and then the average mass of a whale in the populations. The average mass was multiplied by the abundances to estimate the pre-whaling and modern biomass. The age-structured models were then used to estimate the biomass (expressed as tons of carbon yr^−1^ ind^−1^) of carcasses of each species produced per individual in the species, termed the gross flux. Multiplying by the abundance values by the gross flux and dividing by 2 gives an estimate of the flux (tons carbon yr^−1^) exported from the euphotic zone by each species.

The accounting above only considers the impact of whaling on the carbon stored in whale populations. The responses of marine ecosystems to fishing and whaling are complex and highly variable. Strong top-down effects have been reported in some ecosystems [14], [15], [16], although alternative explanations may apply even in some of the most cited examples [17], [18]. Even in the Southern Ocean, the response of the ecosystem following intense whaling is not clear and was complicated by physical changes [19], [20], [21]. On large spatial and long time scales, the most likely response to the reduction of one species is an increase in its competitors. For ecosystems heavily impacted by whaling, this means an increase in smaller species as has been observed in the Pacific [22] and in the Ross Sea [19].

**Figure 2:**
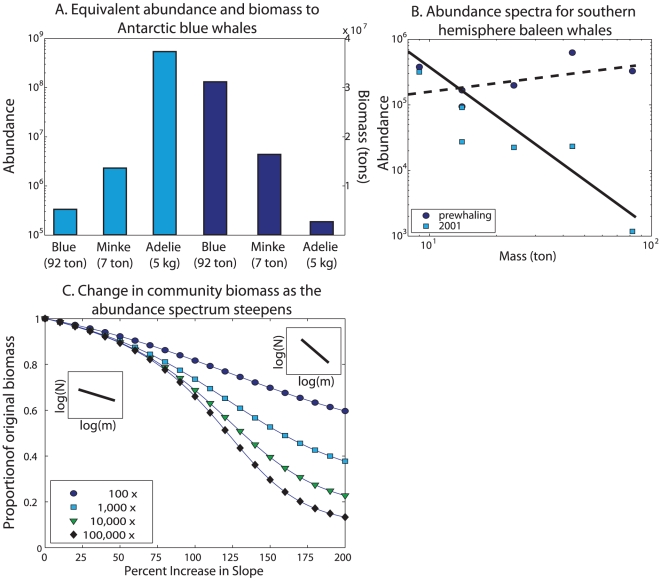
Consequences of reducing the abundance of large species on abundance and biomass. a) Abundance (light blue, scale on left) and biomass (dark blue, scale on right) of blue whales lost from the Southern Ocean. Based on metabolic scaling, the same amount of food could support larger populations of minke whales or penguins, but the biomass of these populations would be less than the original blue whale population. b) Abundance spectra for southern hemisphere mysticetes before whaling (dark circles) and in 2001 (light squares). The regression line for the pre-whaling spectrum (dashed line) has a slope and 95% confidence bound of −0.36±1.01, while the 2001 spectrum (black line) has a slope of 2.05±1.30 and the regression is significant (p<0.05, r^2^ = 0.83, n = 6). c) Impact of steepening the abundance spectra on the total biomass contained in four different communities. In all cases, the food requirements of the community were kept constant as the slope varied. The communities differ in the range of masses contained in the community: 2 (circles), 3 (squares), 4 (triangles), and 5 (diamonds) orders of magnitude.

Unlike the carbon stored in trees, carbon stored in animal tissue must be constantly maintained by feeding. The rate, *R*, at which carbon is respired by an animal depends on its mass, *m*, raised to a power *α*:
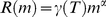
where *γ(T)* is a temperature dependent coefficient. Values of *α* near 3/4 have been found for a wide range of organisms [23], [24]. This relationship means that larger animals require less food per unit mass and thus, they are more efficient at storing carbon than smaller animals [25]. The amount of krill that supported the 3.3×10^5^ blue whales lost from the Southern Ocean could support 2.2×10^6^ minke whales (7 tons each) or 5.2×10^8^ penguins (5 kg each) (Figure 2a). However, the biomass in these populations would be only 50% or 8%, respectively, of the biomass of the missing blue whales.

The calculations represented in Figure 2a envision the complete replacement of one population by another. A more likely scenario is that the food that went to the removed individuals is spread over a range of species or size classes. Marine communities tend to be strongly size structured, and over a large range of masses, the abundance, *N*, tends to follow a power law:
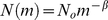
where *N_0_* is a scaling constant [26], [27]. For a community feeding on the same food source, the slope of the spectrum, *β*, should be near 3/4 [22]. The total carbon consumed by the community per unit time (*R_tot_*) is then the product of the abundance spectrum and *R*(*m*) from above:
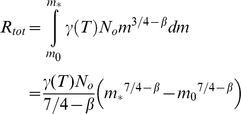
using *α* = 3/4. Communities that are strongly fished tend to have steeper abundance spectra (larger β), and the size distribution of baleen whales in the Southern Ocean supports this view. Prior to whaling, β was not significantly different from zero, while in 2001, β was nearly 2 (Figure 2b). The latter value is comparable to other heavily-exploited ecosystems such as the North Sea [28]. Assuming total consumption by the community remains the same, steepening the size spectrum will lead to an increase in the number of organisms in the community but a decrease in biomass. For the community of baleen whales with masses spanning two orders of magnitude, increasing β from 3/4 to 2 (a 166% increase) results in a 30% decrease in biomass (Figure 2c). In the Southern Ocean, the species that consume Antarctic krill range from penguins to blue whales. For a community with a 5 order of magnitude range, steepening the spectrum reduces the total biomass by more than 70%. In other words, the same amount of primary productivity can support a higher biomass of large individuals due to the increase in metabolic efficiency with increasing size.

The direct removal of carbon by whaling and fishing, coupled with the steepening of the size spectra mean that marine ecosystems now store less carbon than they once did. In addition, the reduction of the populations of large vertebrates also altered how carbon is transferred from one ecosystem to another in the ocean [29]. From a carbon-cycle perspective, the most interesting movements of carbon are those from the euphotic zone to the deep ocean which can sequester carbon for hundreds to thousands of years [1]. Whale falls are the most well-studied example of this large vertebrate flux and are common enough that communities of organisms have adapted to exploit this resource [30], [31]. Using estimates of current population sizes, we calculate that the total carbon flux from 8 baleen whale taxa is currently 2.8×10^4^ tons C yr^−1^ (Table 1). Using estimates of pre-whaling abundance, the total flux would be nearly an order of magnitude greater, or 1.9×10^5^ tons C yr^−1^, a value consistent with earlier estimates [32]. Genetic work suggests that pre-whaling populations may have been a factor of 10 larger than indicated by catch records [6]. These estimates provide an upper-bound on the pre-whaling flux of 1.9×10^6^ tons C yr^−1^, or 0.1% of the ocean's net carbon sink [33]. Although less established than whale falls, non-predation deaths of tuna, billfish, sharks, and other large pelagic fish should also contribute to a flux of organic carbon out of the euphotic zone. As with biomass, an increase in smaller competitors could compensate for some of the lost carbon flux; however, since smaller animals have higher predation rates, much of the potential flux will be lost to consumption.

**Figure 3:**
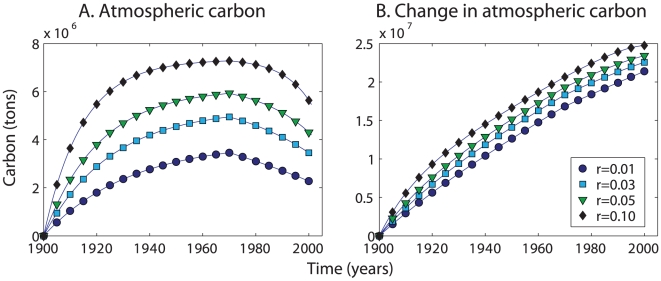
Carbon footprint of 20th Century whaling. A. Total carbon in the atmospheric carbon pool. Each line corresponds to a different biomass accumulation rate (*r*) as in Figure 1. Whales killed by whaling where added to the atmospheric pool. A proportion of the population in each year was assumed to die and sink. This export flux was removed from the atmospheric pool. B. Difference in atmospheric carbon with and without whaling.

By combining the estimates of carbon export with those for whale biomass, we estimated the total carbon footprint of whaling. For this calculation, we transferred the carbon removed by whaling into a pool that we call the atmosphere (Figure 3a). We recognize that how and when the carbon in a killed whale reaches the atmosphere will depend on the manner in which it was processed and the products that were produced from its carcass. We also used the flux estimates described above to compute the carbon exported by the whale population in each year. The exported carbon was removed from the atmospheric pool. We also computed an undisturbed atmospheric pool by removing the carbon exported by the 1900 population for each year. The total carbon footprint of whaling is then the difference between the atmospheric pool with and without whaling (Figure 3b). Assuming a recovery rate (*r*) of 3% gives an estimate of 2.35×10^7^ tons C added to the atmospheric pool by whaling.

## Discussion

The carbon stored in populations of marine vertebrates is only a small part of the total carbon in marine ecosystems; however, the impact of rebuilding stocks of fish and whales would be comparable to existing carbon sequestration projects. For example, rebuilding the southern hemisphere blue whale population would sequester 3.6×10^6^ tons C in living biomass. Assuming 82 tons C ha^−1^ of forest [34], the new blue whales would be equivalent to preserving 43,000 hectares of temperate forest, an area comparable in size to the City of Los Angeles. Rebuilding all of the whale populations in Table 1 would store 8.7×10^6^ tons C, equivalent to 110,000 hectares of forest or an area the size of the Rocky Mountain National Park. As a population nears its carrying capacity, the rate at which it is accumulating carbon slows, but even at carrying capacity, marine vertebrate populations can still export carbon through sinking carcasses. If restored to pre-industrial levels, southern hemisphere blue whales would remove 70,000 tons C yr^−1^ through sinking of dead whales. Restoring all whale populations would export an additional 160,000 tons C yr^−1^. This flux would be equivalent to preserving 843 hectares of forest each year.

Ocean iron fertilization is the most widely discussed idea for sequestering carbon in the ocean, and our calculations suggest that rebuilding whale and fish populations would compare favorably with these schemes. Iron fertilization schemes revolve around the observation that primary productivity in large areas of the ocean is limited by the availability of iron [2]. If iron is added to these regions, then phytoplankton should bloom and carbon should be exported through the biological pump. In theory, it should be possible to add a few hundred tons of iron sulfate to the Southern Ocean and sequester millions of tons of carbon [35]. While several experiments have demonstrated the iron limitation hypothesis, these experiments have had mixed success at stimulating export. The most successful experiment, in terms of the measured carbon export, exported a maximum of 900 tons C [36]. At these rates, it would take 200 such blooms each year to match the export potential of fully restored whale populations. Given that larger experiments have produced little or no export [37] and that iron fertilization could have unintended consequences such as increases in toxic species [38], [39], conserving populations of large marine vertebrates may represent a more ecologically sound alternative.

The main disadvantage to using populations of large vertebrates as a carbon management tool is that, while humans may be responsible for their low population levels, there are often limited options for accelerating the rebuilding of these populations. For commercial fish species, reducing fishing mortality is a necessary step; however, as evidenced by the slow recovery of Newfoundland cod, reducing fishing does not guarantee a recovery [40]. Hunting of baleen whales has largely ceased. The greatest threats to current whale populations are likely mortality due to ship strikes [41] and potentially reduced food supplies due to climate variability or competition with humans [42]. A better accounting for the potential of these species to sequester and export carbon could allow organizations to claim carbon credits for actions that support the rebuilding of these populations.

Our calculations add to a growing body of literature on the importance of large organisms to the ocean's biogeochemical cycles. In addition to storing and exporting organic carbon, fish can export inorganic carbon through the excretion of calcium carbonate [10], while whales and krill in the Southern Ocean may help retain iron in the surface waters [11]. Our analysis also suggests that marine ecosystems with larger individuals or larger species could support a higher biomass due to the increase in metabolic efficiency with body size. Due to the strong relationship between fecundity and body size in fish [43], [44], a community with a fully populated size spectrum would have a higher reproductive output and would likely be more resilient than a population with a truncated size spectrum. These calculations suggest that conserving larger species and largest individuals within species should be a top conservation priority.

## Materials and Methods

### Part 1. Whale populations before and after whaling

Our study builds from Christensen's comprehensive assessment of world-wide whale populations [12]. She estimated both the pre-whaling abundance and the modern (2001) values. As discussed above, estimates of pre-whaling abundance based on catch records could underestimate the true pre-whaling abundances due to underreporting. Independently assessing the accuracy of the pre-whaling abundance estimates is exceedingly difficult. Population genetic techniques are one approach, and these analyses consistently yield pre-whaling levels much higher, up to a factor of 10, than stock assessment-based methods [6]. Although considerable uncertainly exists surrounding the pre-whaling estimates we use, these values represent conservative estimates of the population sizes.

Right (*Eubalaena glacialis*, *E. japonica*, and *E. australis*) and bowhead (*Balaena mysticetus*) whales were included in the population and biomass calculations. However, these species were treated separately in calculations of vertical flux and whaling as discussed below. Christensen did not estimate the abundance of southern hemisphere right whales. For this population, we used estimates from [45].

We excluded sperm whales (*Physester macrocephalus*) from our calculations. This species tends to feed at great depth. The prey available at these depths likely derives a portion of its nutrition from the organic matter sinking from the photic zone. Thus, by feeding on these animals and returning to the surface to respire, sperm whales could potentially counteract the export of carbon which we aim to calculate. Determining whether sperm whales represent a net upward or downward flux of carbon is an interesting calculation, but one that is beyond the scope of this study.

To estimate the flux of carbon from whale populations, we must first estimate the number of whales that die each year. For simplicity, we will consider a generic whale population with general mysticete characteristics. Our model is a simple age structured model, and we assume that the population is at steady state with a total population of *K*. We divide the population into age classes of size one year. We assume that whales do not live longer than *n* years. Studies of populations of large baleen whales suggest that females become sexually mature between 5–10 years [13], [46], [47], [48]. All baleen whales have a well-defined annual reproductive cycle, with pregnancy lasting the majority of a year followed by several months of nursing. This sets the absolute maximum calf production rate at 0.5 births female^−1^ yr^−1^. The actual production rate will be lower, as females do not always become pregnant immediately following a birth.

Whales have few predators and their natural mortality rates are very low [49], [50]. Demographic studies of modern whale populations generally assume a constant mortality rate for all age classes and then add an additional mortality term for calves [13], [49], [51] We will use this simple formulation and solve for *s* (non-calf) and *s*_1_ (calf) survival rates that balance the prescribed fecundity schedule:
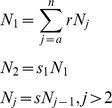
(1)where *r* is the fecundity (births per individual), *a* is the age at which females become mature, and *n* is the maximum age. We can remove the recursion:
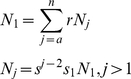
(2)producing an explicit function for all ages except *N_1_*.

For a population to be at steady state, the age-dependent fecundity must be balanced by mortality:
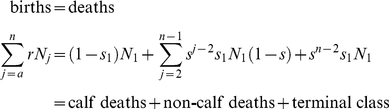
(3)Introducing the expression for *N_j_* into equation (2):
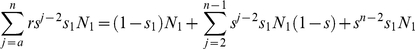
We can remove *s_1_N_1_* from both sides:
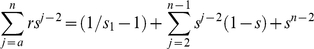
and then split the summation on the right:
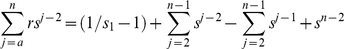
We can incorporate *s_n−2_* into the first summation on the right by adjusting the limit of the sum:
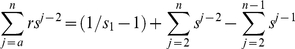
To make things simpler, we adjust the limits of the summations so that both on the right involve powers of *j*:
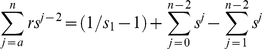
It is then easy to see that most of the terms cancel, leaving only *1/s_1_* on the right hand side. This simplifies to
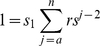
We then apply the identity:
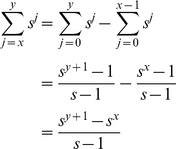
to the remaining summation:
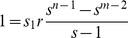
(4)


Equation 2 has five parameters: *r*, *s_1_*, *s*, *m*, and *n* that define the population dynamics of our simple model. If the identity in (4) holds, then the population will be in steady-state, the presumed condition before whaling. Specific values of these parameters are not known, but the ranges are generally well established (Table 2). We employed a Monte-Carlo procedure to find a range of plausible parameter combinations. We created 10,000 populations (combinations of the five parameters) by picking a value for each parameter from a normal distribution. The means of the distributions were assumed to be the midpoint of the range in Table 2, and the standard deviation was assumed to be half of the range. For each population, we applied equation (4) to find a new value for each parameter while leaving the other four parameters fixed that produced a steady-state population. Thus, we created 50,000 steady-state populations. These were ranked based on the likelihood of selecting each combination from the normal distributions. We then used the 1,000 most-likely populations to estimate the mean parameter values for each species (Table 3).

**Table 2:** Parameters ranges input into the demographic model.

	Age at Maturity		Maximum Age		Calving Interval		Juvenile Survival		Adult Survival	
Species	Min	Max	Min	Max	Min	Max	Min	Max	Min	Max
Blue	5	10	110	150	2	10	0.600	0.910	0.915	0.990
Fin	6	10	90	150	2	10	0.600	0.910	0.915	0.990
Humpback	4	10	48	100	2	10	0.600	0.910	0.915	0.990
Sei	8	11	65	120	2	10	0.600	0.910	0.915	0.990
Bryde's	7	12	50	120	2	10	0.600	0.910	0.915	0.990
Minke	6	10	40	80	2	8	0.600	0.910	0.915	0.990
Gray	5	9	75	120	2	10	0.600	0.910	0.915	0.990
Right	6	10	75	120	3	10	0.600	0.910	0.915	0.990
Bowhead	8	20	100	200	3	10	0.600	0.910	0.915	0.990

**Table 3:** Demographic parameters for steady state populations.

	Age at Maturity		Maximum Age		Calving Interval		Juvenile Survival		Adult Survival	
Species	Mean	SD	Mean	SD	Mean	SD	Mean	SD	Mean	SD
Blue	7.5	1.5	129.8	11.5	8.4	5.9	0.751	0.092	0.955	0.020
Fin	8.0	1.2	118.2	18.4	8.5	6.0	0.754	0.091	0.956	0.020
Humpback	6.9	1.7	74.3	14.4	7.7	3.7	0.756	0.086	0.957	0.019
Sei	9.5	0.9	91.6	16.4	7.7	4.9	0.751	0.093	0.958	0.019
Bryde's	9.5	1.4	81.4	20.7	7.3	4.0	0.755	0.090	0.958	0.018
Minke	9.0	1.2	86.2	8.0	6.8	4.0	0.750	0.093	0.954	0.019
Gray	7.0	1.2	97.3	14.0	8.2	5.1	0.746	0.096	0.956	0.020
Right	8.1	1.2	96.8	12.1	7.8	4.0	0.758	0.090	0.957	0.017
Bowhead	14.0	3.5	147.2	29.9	7.7	6.2	0.761	0.089	0.960	0.018

The population parameters define a steady-state age structure. In order to estimate the population biomass and carbon flux, we need to know the mass of a whale of a certain age. Lockyer [52] fit von Bertalanffy weight-at-age relationships for blue, fin, and sei whales. Using these results and additional growth and longevity information [53], [54], [55], we established models for each species and applied the models to the age distributions to compute an average mass for each species (Table 4). Longevity information was especially hard to come by. In our model, whales that survive past the maximum age are removed; thus, we seek the maximum age possible for each species. When possible, we used the maximum reported age as the lower bound for our calculations and then specified a proportionally higher upper bound.

**Table 4:** Parameters for von Bertalanffy mass-at-age model and carbon export potential.

	Male			Female			Export (tons C yr^−1^)		
Species	m_max_	k	a_0_	m_max_	k	a_0_	Min	Mean	Max
Blue	102.0	0.2	4.9	117.0	0.2	4.5	0.120	0.424	0.729
Fin	55.0	0.2	5.3	64.5	0.2	4.8	0.056	0.223	0.391
Humpback	30.0	0.1	9.4	30.0	0.1	9.4	0.033	0.103	0.172
Sei	18.0	0.1	9.4	19.5	0.1	10.0	0.018	0.062	0.105
Bryde's	18.0	0.1	9.4	19.5	0.1	10.0	0.020	0.061	0.102
Minke	6.0	0.2	1.0	6.0	0.2	1.0	0.008	0.018	0.028
Gray	30.0	0.1	9.4	30.0	0.1	9.4	0.031	0.105	0.179
Right	40.0	0.1	9.4	40.0	0.1	9.4	0.051	0.137	0.224
Bowhead	40.0	0.0	22.0	40.0	0.0	22.0	0.028	0.051	0.074

Using the mortality terms (*s* and *s*_1_), we can estimate the number of whales lost from each age class. We can then apply the weight-at-age functions to convert the number of whales dying into tons of dead whales. Using the proportions of bone, lipid, and protein in Jelmert & Oppen-Berntsen [32], we can convert the total biomass into tons of carbon. By normalizing to a population of size 1, we have the gross flux in terms of carbon lost from each population per year per whale in the population. We computed the flux for females and males using the 1,000 most likely parameter configurations for each of the 8 populations. We then computed the mean flux for each population, as well as the 95% confidence limits (Table 4).

Smith and Baco [30] estimate that up to 90% of whale mortalities become whale falls. We used their most conservative estimate and assumed that 50% of mortalities (or equivalently 50% of each carcass produced) reach the seafloor. The balaenid species (right whales–*Eubaleana* spp. and bowhead whales–*Balaena mysticetus*) present a special problem for sinking fluxes. These whales were dubbed “right” by whalers due to their high quantities of blubber and their tendency to float when killed. We expect that most natural whale deaths would be caused by disease or starvation. These whales would tend to be in poor condition and more likely to sink. We thus assumed that 10% of mortalities resulted in export. While we believe the proportions used for all whales are conservative, these numbers have not been measured. Combining the population estimates with the fluxes, we can produce the expected pre- and post- whaling carbon fluxes (Table 4).

### Part 2: Biomass removed by whaling

For this section, we compute the total impact of whaling during the 20th C in terms of carbon storage and carbon flux. For this calculation, we excluded right and bowhead whales as these species were fully exploited prior to the 20th C [12]. We began by assuming that the pre-whaling abundances (and the implied biomasses) for the non-balaenid species computed by Christensen represented conditions in 1900. We assumed that whale populations accumulate biomass at a steady rate *r*. In reality, the biomass accumulation rate should be slower when whale biomass is high, reflecting density dependence. However, the impact of this change is small. We further assumed a constant exploitation rate for the period 1900–1970. To compute the exploitation rate, we worked backward from the 2001 biomass to estimate the biomass in 1970 using *r*. Then, we computed the exploitation rate required to reach the 1970 biomass from the 1900 value. We performed these calculations for four values of *r*: 1, 3, 5, and 10% yr^−1^ (Figures 1 and 3). We presented the 3% calculations in the main text to add an extra measure of caution to the calculations.

### Part 3: Body size and carbon storage

We start by assuming an underlying power law relationship between abundance and size (mass):
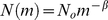
The energy (*R*) required by all individuals of size *m* in the community is then the product of *N*(*m*) and the metabolic rate:
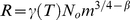
using the standard 3/4 scaling for metabolism and where *γ*(*T*) is a temperature dependent coefficient on metabolic rate. The total of carbon consumed by a community is then proportional to the integral of *R(m)* between the smallest (*m_0_*) and largest (m_*_) sizes:
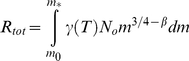
Assuming *β*≠0.25:
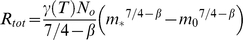
(5)Assuming the total carbon consumed does not change, then changing the spectral slope by a factor *q* leads to a change in *N_0_*:
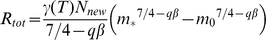
(6)Setting (5) equal to (6) and *N_new_ = rN_0_*, we can solve for the proportional change *r*:
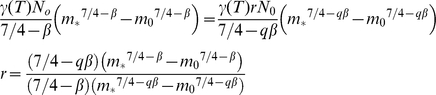
(7)The total biomass in the new community is now:
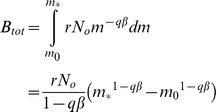
(8)The new biomass is a factor *f* times the original biomass:
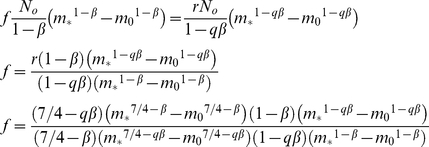
(9)


### Part 4: Size spectrum in southern ocean mysticetes

Christensen's original analysis of pre and post whaling populations distinguished between whale populations in the North Atlantic, North Pacific, and southern hemisphere. We used logarithmic regression to fit the slope (*β*) and intercept (*N_0_*) of the abundance spectrum:
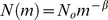
for the three regions, before and after whaling. Significant relationships were only found for the southern hemisphere (Figure 2b). Before whaling, the slope of the abundance spectrum (*β*) was not significantly different from 0, while in 2001, the slope was 2 (p<0.05, r^2^ = 0.83, n = 6).
